# MRI/MRS in neuroinflammation: methodology and applications

**DOI:** 10.1007/s40336-015-0142-y

**Published:** 2015-09-10

**Authors:** Mario Quarantelli

**Affiliations:** Biostructure and Bioimaging Institute, National Research Council, Via T. De Amicis 95, 80145 Naples, Italy

**Keywords:** Neuroinflammation, Magnetic resonance imaging, Magnetic resonance spectroscopy, Superparamagnetic iron oxides

## Abstract

Neuroinflammation encompasses a wide range of humoral and cellular responses, not only enabling the CNS to fight various noxious events, including infections and trauma, but also playing a critical role in autoimmune as well as in neurodegenerative diseases. The complex interactions of immune, endothelial, and neuronal cells that take place during inflammation require an equivalent complexity of imaging approaches to be appropriately explored in vivo. Magnetic Resonance provides several complementary techniques that allow to study most mechanisms underlying the brain/immune interaction. In this review, we discuss the MR approaches to the study of endothelial activation, blood–brain barrier permeability alterations, intercellular compartment modifications, immune cell trafficking, and of metabolic alterations linked to immune cell activity. The main advantages and limitations of these techniques are assessed, in view of their exploitation in the clinical arena, where the complementarity of the information that can be obtained has the potential to change our way of studying neuroinflammation, with implications for the management of several CNS diseases.

## Introduction

Neuroinflammation plays a critical role in the acute phases of neuronal damage (e.g. to fight infections, and to remove post-apoptotic cell debris), as well as in the subsequent repair processes, including neurogenesis, oligodendrogenesis, and axonal sprouting [1].

In full-blown neuroinflammation, the activation of microglia, the brain-resident macrophages that are the initial responders to tissue damage, is generally accompanied by blood–brain barrier (BBB) breakdown, release of biohumoral inflammation mediators, such as cytokine and chemokine, and by blood-borne leukocyte infiltration. These phenomena result in the release, in the local microenvironment, of oxidative and nitrosative products by machrophages, microglia and astrocytes, and in the production of excitotoxic metabolites, causing tissue damage.

Beside intense inflammatory reactions, such as those that accompany stroke or acute inflammatory conditions (e.g. multiple sclerosis relapses), also a prolonged, milder inflammatory response, mainly associated with microglial activation, can result in a damage to the brain cells, which in turn can promote a reactivation of neuroinflammation, providing a possible basis for the reciprocal reinforcement of neuroinflammatory and neurodegenerative phenomena [2].

Of note, neuroinflammation seems also to accompany the neuronal activity under physiological conditions, representing a tool to modulate the metabolic demands in response to the physiological variations of neuronal network activity. Indeed, the term “neurogenic inflammation”, classically associated with the capacity of sustained neuronal activity to trigger inflammatory reactions in peripheral tissues [3], has recently been extended to encompass the inflammatory reactions within the CNS that can be triggered by similar mechanisms [4].

Neurogenic neuroinflammation is postulated to have a physiological role, possibly allowing the CNS to cope with increased metabolic demands deriving from increased computational activities, or promoting regeneration. However, an excessive duration of sustained neuronal activity (such as in chronic pain or in psychological stress) or an excessive extent of sustained stimulation (such as in epileptic seizures) may render this mechanisms maladaptive, resulting in neuronal damage.

**Fig. 1:**
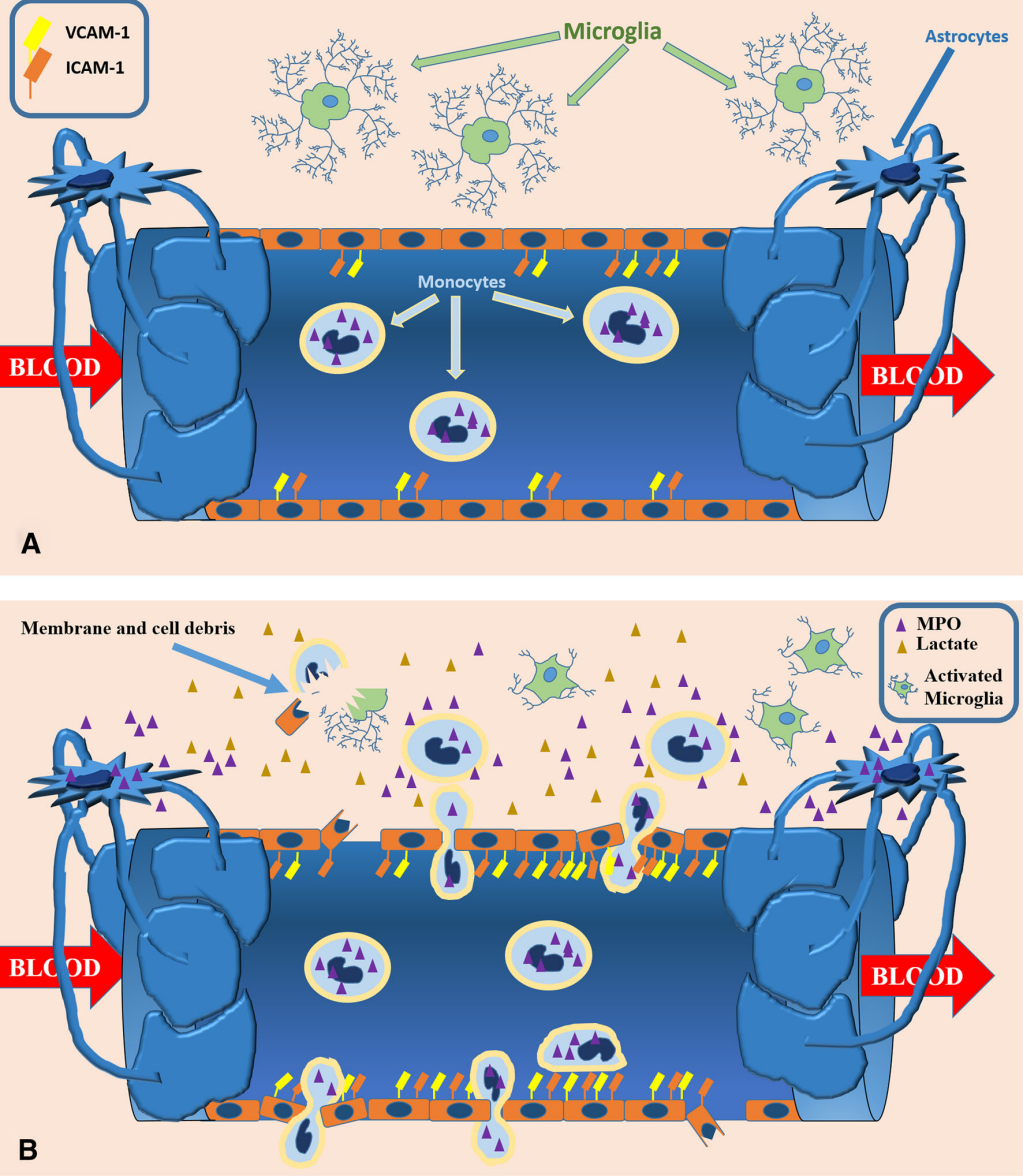
Main neuroinflammatory mechanisms probed by MRI. Modifications of the neurovascular unit include alterations of the BBB permeability, associated with overexpression of adhesion molecules, which induce blood-borne monocytes to arrest and crawl along the endothelium, crossing it by diapedesis along loosened intercellular junctions. Machrophage and microglia activation results in increased MPO activity and lactate accumulation in the interstitium, along with the presence of cellular debris resulting from cell damage due to oxidative and excitotoxic phenomena

Several aspects of neuroinflammation can be studied by MRI (Fig. 1), using a heterogeneous host of approaches (Table 1) that allow to probe the mechanisms underlying the brain/immune interaction at the level of vascular, cellular, and interstitial compartments.

**Table 1:** Main neuroinflammatory phenomena that can be assessed using MRI and/or MRS, with corresponding study techniques and stage of development of the technique

Neuroinflammatory phenomena	MRI/MRS technique	Development stage
BBB permeability	Dynamic contrast-enhanced MRI	Clinical
Endothelial activation	USPIO-labelled antibodies against surface molecules	Investigative
Blood-borne cell migration	Iron oxide colloids to track phagocytic cells	
	Non-specific labeling	
	In-vivo labeling	Pilot clinical studies
	Ex-vivo labeling	Preclinical
	Iron oxide-labeled antibodies against immune cells epitopes	Investigative
Myeloid/glial cells activation	Myeloperoxidase-activated contrast media	Investigative
	Spectroscopy → Creatine	Clinical
	Spectroscopy → Myoinositol	Clinical
Interstitial modifications		
Macromolecular composition	Magnetization transfer	Clinical
Lactate accumulation	Spectroscopy → Lactate	Clinical
Cell debris accumulation	Spectroscopy → Choline (cell membranes turnover)	Clinical
Cell debris accumulation	Spectroscopy → Lypids/macromolecules	Clinical
Vasogenic edema	Diffusion → ADC/FA	Clinical
Sequelae		
Demyelination	Diffusion → Radial diffusivity	Clinical
Neuronal loss	Spectroscopy → NAA (neuronal loss/dysfunction)	Clinical
Axonal loss	Diffusion → Axial diffusivity	Clinical
Axonal loss	Conventional MRI segmentation → Black holes load	Clinical
Demyelination/gliosis	Conventional MRI segmentation → T2w lesion load	Clinical

*Investigative* preliminary proof-of-concept studies have demonstrated in vivo sufficient sensitivity and specificity, *Preclinical* used in preclinical studies to monitor disease progression and/or drug effect in neuroinflammatory conditions, but no human studies have been carried out, *Pilot clinical studies* preliminary human studies are available, *Clinical* routinely used as a clinical tool, or employed as a biomarker in clinical trials

In this review, MRI methods that have been applied to assess neuroinflammatory phenomena in preclinical and clinical studies are described, grouped according to the probed neuroinflammatory processes. MRI methods that study endothelial activation, BBB permeability alterations, intercellular compartment modifications, and immune cell trafficking are thus reported. In addition, MR spectroscopy (MRS) markers of glial activation and of immune cell activity, suitable to monitor neuroinflammatory phenomena, are summarized.

For each technique, the main advantages and limitations are discussed in view of their potential clinical exploitation.

## BBB breakdown

The study of BBB integrity represents the most diffuse approach to the assessment of the immune response in the brain using MRI, given the ready availability in clinical use of contrast agents that can effectively probe it.

It should be here noted that, although BBB permeability alterations may in principle not be needed to define the presence of neuroinflammation [5], most neuroinflammatory stimuli primarily affect BBB integrity, and when any of the several BBB constituents fails, almost invariably neuroinflammation and neurodegeneration ensue [6].

Consequently, although the assessment of BBB permeability does not detect/monitor neuroinflammation “per se”, it represents a sensitive clinical tool in pathologies with neuroinflammatory components.

Alterations of BBB, ranging from hyperpermeability to a widespread disruption of tight junction complex assembly [7], allowing blood-born leukocytes extravasation [8], occur in several physiological and pathologic conditions. Foci of complete BBB disruption are generally present in infectious diseases, such as in meningitis and encephalitis [9], or in the active phases of multiple sclerosis (MS) [10]. However, milder permeability changes, not associated with leukocyte trafficking, can also occur in pathologies not primarily linked to neuroinflammation, such as cerebral small vessel disease, diabetes [11], Alzheimer’s disease [12], or even in association with normal aging [13], possibly as a consequence of subtle chronic neuroinflammatory phenomena that accompany these conditions.

Contrast-enhanced MRI (CE-MRI), based on the use of contrast media containing gadolinium, is the most commonly used non-invasive imaging method to assess BBB alterations in both clinical and preclinical studies [14].

CE-MRI takes advantage of the intravascular compartmentalization in the brain, under normal conditions, of Gd-chelates, which do not pass the intact BBB. In clinical settings, CE-MRI is generally performed by acquiring T1-weighted images several minutes following i.v. administration of contrast medium, to reveal regions of reduced T1 (appearing bright on T1-weighted images) due to contrast media extravasation in the presence of BBB breakdown.

Although very recent reports of signal changes in deep gray matter structures may indicate a long-term retention of Gd from previous MRI CE-MRI studies [15, 16], at least when using linear molecules [17, 18], Gd-chelates in clinical use have a very favorable safety profile, allowing for repeated administrations, at least in patients with normal renal function.

**Fig. 2:**
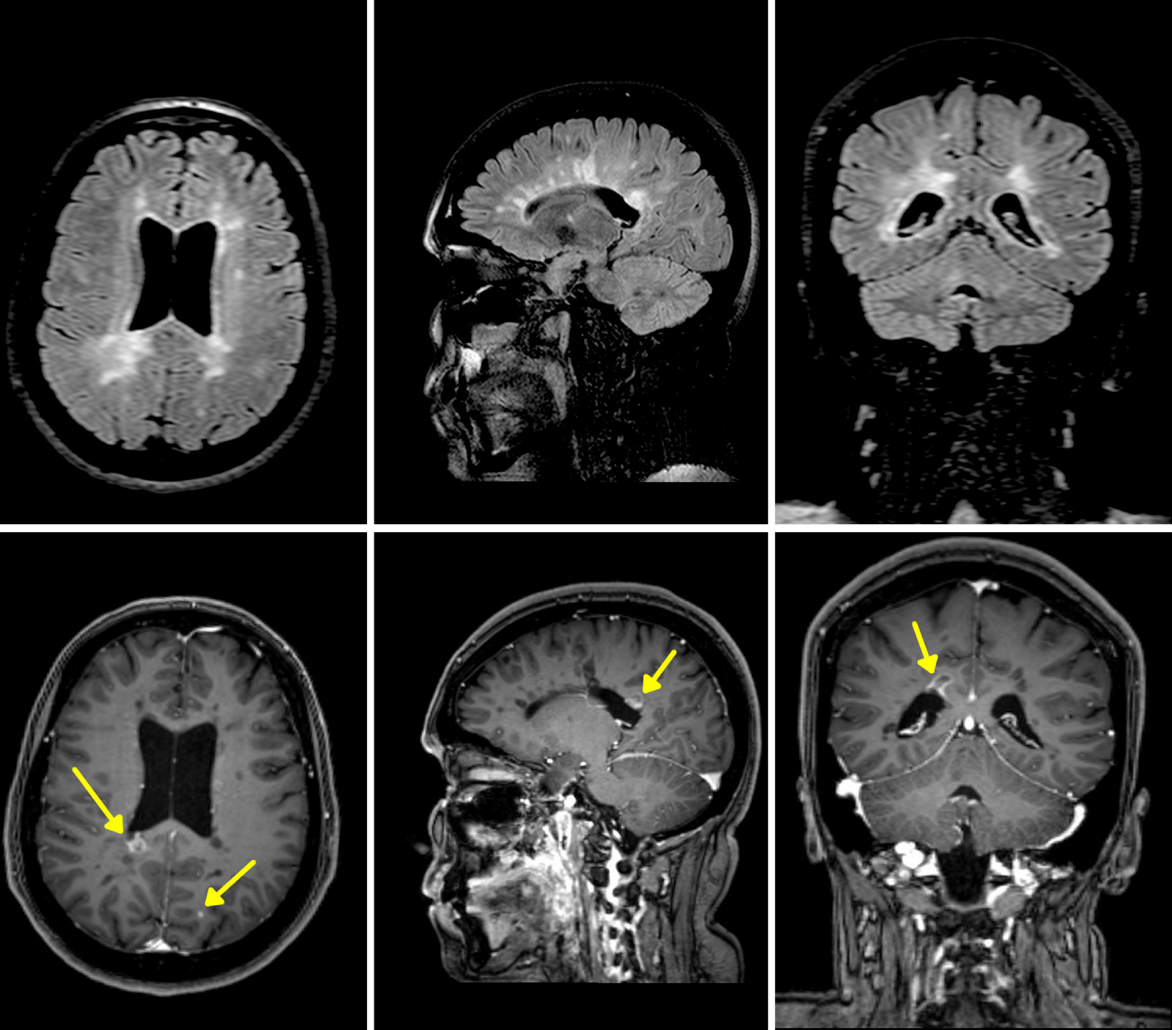
Axial, sagittal and coronal MRI slices (3 Tesla, Philips Medical Systems, Eindhoven, The Netherlands) from a 3D FLAIR sequence (*upper row*) and the corresponding sections from a post-Gd T1-weighted volume (*bottom*), in a multiple sclerosis patient. A right posterior paraventricular lesion shows peripheral incomplete rim of contrast enhancement, consistent with BBB breakdown due to active inflammatory phenomena. A punctate iuxtacortical left occipital enhancing lesion is also present

A typical application of CE-MRI is the monitoring of inflammatory activity in MS (Fig. 2). The possibility to simultaneously assess ongoing inflammatory activity (by contrast-enhancement detection), along with accumulated lesion load (by T2w lesion segmentation) and neuronal loss (by segmentation of normal brain tissues) [19], has rendered conventional CE-MRI the mainstay in clinical trials as surrogate biomarker for monitoring treatment response in MS.

Note that segmentation methods for measurement of MS lesion load and brain atrophy, although linked to sequelae of inflammatory phenomena, are not being discussed in this review, which is focused on methods that target active inflammatory phenomena.

Alternatively, dynamic acquisition of MR sequences sensitive to Gd concentrations, during the i.v. bolus administration of contrast media (dynamic CE-MRI), coupled with appropriate modeling strategies, has been used to quantitate regional vessel permeability, assessing cerebral blood flow and BBB permeability simultaneously [20, 21].

Different algorithms have been developed to assess the trans-capillary leakage of the contrast media molecule, typically estimated in terms of forward volumetric transfer constant (*K* ^trans^), which is, however, also affected to a variable extent by blood flow [22].

To disentangle blood flow and vessel permeability, allowing to measure selectively the trans-capillary permeability in terms of Permeability-Surface area product (PS), several methods have been developed, which take advantage of the simultaneous measurement of CBF and *K* ^trans^ allowed by DCE-MRI, provided sufficient temporal and spatial resolution are obtained with appropriate sequences. These methods are based on the use of a more complex modeling (e.g. adiabatic approximation to the tissue homogeneity [23]), coupled to a two-compartment exchange model [24], and have not found large application, partly because of the requirements they impose on scanner hardware. More recently, it has been proposed to incorporate in the modeling the blood flow measured using arterial spin labeling (an MRI technique for CBF measurement available on most scanners, which can be acquired prior to the DCE-MRI acquisition), to calculate flow-corrected PS maps [25].

In addition to post-processing differences, variability of scanner/sequence performance and of acquisition parameters contribute to a lack of comparability of permeability values obtained in different centers. Consequently, despite the relative maturity of dynamic CE-MRI methods, and the potential interest of permeability measures to monitor neuroinflammation, the heterogeneity of the approaches currently hinders inter-study comparability, thus limiting the impact of available results and their utility to understand the role of vessel permeability alterations in disease pathophysiology [20, 26].

## Adhesion molecules

Adhesion, the first step in the migration of blood-borne leukocytes to the inflammation site, is mediated through interaction of leukocyte integrins with molecules exposed by endothelial cells, such as selectins (e.g., P- or E-selectin), or adhesion molecules including vascular cell adhesion molecule 1 (VCAM-1), intercellular adhesion molecule 1 (ICAM-1), or platelet endothelial cell adhesion molecule 1 (PECAM-1). Following adhesion, monocytes transmigrate through the vessel wall by diapedesis and migrate along cytokine gradients toward the site of inflammation [27].

Antibodies against several of these adhesion molecules have been conjugated to superparamagnetic particles of iron oxide, which exhibit strong T1 and T2* relaxation effects, typically stronger than Gadolinium chelates [28], and can be visualized by suitable MR sequences.

Following different chemical routes for the synthesis [29], superparamagnetic iron oxide nanoparticles of a large range of sizes can be obtained, which affect their biodistribution and relaxometric properties. In addition, as these properties are largely conditioned by the effective size of the particles (which in turn varies depending on their hydratation state [30]), the hydrodynamic diameter is generally considered when classifying these colloids.

Accordingly, iron oxide particles can be divided into four subgroups (modified by [31]):

- Monocrystalline Iron Oxide particles (MNIO, hydrodynamic core <20 nm)
- Ultrasmall SuperParamagnetic Iron Oxide particles (USPIO, 20 nm < hydrodynamic core < 50 nm)
- SuperParamagnetic Iron Oxide particles (SPIO, 50 nm < hydrodynamic core < 1 μm)
- MicroParticles of Iron Oxide (MPIO 1 μm ≤ hydrodynamic core)

Anti-VCAM-1 antibodies conjugated to 1 μm sized micron particles of iron oxide (VCAM-MPIO) have been demonstrated to specifically accumulate in the site of intracerebral injection of IL-1β [32], as VCAM-MPIO uptake was not present in control animals injected only with normal saline vehicle and was blocked by pre-treatment with anti-VCAM-1 blocking antibodies.

The same approach in the MCAO model of stroke has shown a more extensive area of VCAM-MPIO uptake, compared to diffusion alterations, suggesting that hypoperfused brain regions at risk for infarction upregulate VCAM-1 [33, 34].

Specific uptake of anti-VCAM-1 antibody conjugated to MPIO has been also demonstrated in mice with EAE [35, 36] (above what seen by BBB permeability studies), in a pilocarpin model of seizure [37], in models of vascular dementia [35], and in the APPPS1 model of Alzheimer disease [35].

Other adhesion molecules expressed on endothelial cells, which have been demonstrated to upregulate in presence of inflammation, are the E- and P-selectins (or CD62E/P), which are targeted by sialyl Lewis X (sLex), expressed on leukocytes.

Sufficient sensitivity and specificity for imaging sLex has been achieved using a glyconanoparticle molecule (GNP-sLex), which carries more than 100 sLex molecules on the surface of a dextran-coated USPIO). GNP-sLex has been used to image the expression of endothelial adhesion molecules in MOG-induced EAE and in stroke in rats [38].

ICAM-1, a member of the immunoglobulin superfamily expressed by the endothelial cells, fostering the adhesion of the leukocytes to the endothelium, has also been targeted using anti-ICAM-1 functionalized MPIO, which showed sufficient sensitivity to image neuroinflammation in a MCAO stroke model in vivo [39] and after radiation injury [40].

Finally, both Gd- and USPIO-labeled MRI agents have been developed to target Integrin αvβ3, an adhesion molecule expressed on endothelial cells, macrophages, and platelets [41], although their use has been so far limited to the study of tumor angiogenesis.

Although presently no imaging studies of overexpression of the adhesion molecules in the human brain are available, the efficacy of therapeutic strategies based on the blockage of adhesion molecules in neuroinflammatory diseases, exemplified by the success of this approach in MS [42], is boosting the efforts for translation to the clinical ground of these methods.

However, before the MR imaging of adhesion molecule can gain the clinical arena, several shortcomings must be overcome, including safety issues related to the coating of MPIOs, which are not biodegradable, and for which potential specific side effects (related to the endothelial inflammation triggered by their binding to the target molecules) should also be fully excluded.

## Cellular immune response

The main components of the cellular immune response in the brain are constituted by two groups of cells, both derived from the hematopoietic stem cells through two radically different paths. While the microglia, the resident macrophages of the CNS, originate in the yolk sac, the other mononuclear cells participating in the immune response originate in the bone marrow. Non-microglial mononuclear cells with phagocytic properties involved in the immune response include the perivascular, choroid plexus, and meningeal macrophages, along with the circulating monocytes, which leave the capillaries to reach the CNS tissues under inflammatory conditions [43].

Recent advances in the design of contrast materials currently allow MRI to afford sufficient sensitivity to track the behavior of these cells in vivo, by labeling them with superparamagnetic particles, which provide the possibility to track single cells loaded with single MPIO particles using MRI [44].

In general, the larger SPIO molecules have a shorter half-life in the blood (<10 min in humans [45]) compared to the smaller USPIOs, due to the more efficient removal of these particles from the blood by the reticulo-endothelial system. However, also the coating material and the surface charge of the particles have a significant role in determining their fate and biodistribution, so that the behavior, in terms of labeling efficiency, can still be very different within the same subgroup of molecules, despite their similar size.

**Fig. 3:**
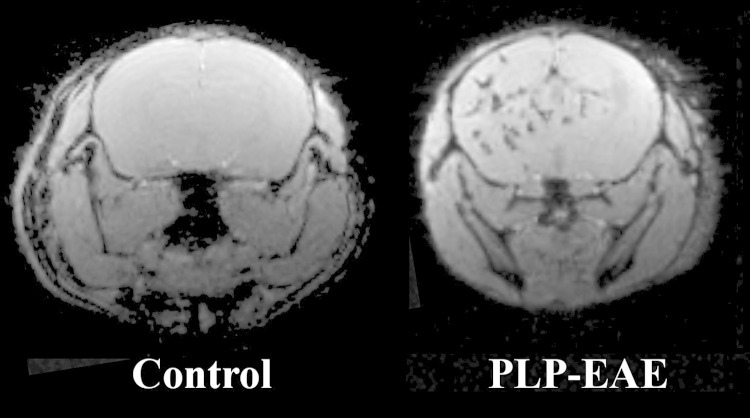
Representative T2*-weighted axial images (9.4 Tesla, Bruker, Billerica, MA) acquired 24 h post-i.v. injection of SPIO (60 nm hydrodynamic diameter, Feraspin-R, Miltenyi Biotec GmbH, Bergisch Gladbach, Germany) in a PLP-EAE mouse, injected at the height of the symptoms, and in a control animal. Multiple foci of USPIO uptake are present in both hemispheric white matter and deep gray matter structures of the EAE model in the right hemisphere

Beside the intrinsic relaxometric properties of the molecules, their effect on the MRI signal intensity is modified by their compartmentalization. In particular, the sequestration of iron nanoparticles in the reticuloendothelial cells dramatically reduces the T1 relaxivity while enhancing their T2/T2* relaxivity [31]. Accordingly, techniques for detecting the cells labeled using these particles have generally focused on gradient-echo sequences, exploiting T2* contrast to allow detection with high sensitivity of their presence in monocytes/machrophages, to track their fate in inflammatory sites, where their accumulation is detected as low-signal foci, owing to T2/T2* shortening (Fig. 3). The high T2* relaxivity of SPIOs, coupled to the use of highly sensitive T2*-weighted gradient-echo sequences, has indeed allowed to track in the mouse brain single cells loaded with SPIOs, following i.v. injection [46].

Different strategies can be adopted to label circulating monocytes with these particles to assess cell trafficking from the blood to the neuroinflammation sites.

Cells can be loaded with iron oxide colloids taking advantage of their natural phagocytic properties, either by incubation of the cells in vitro with the selected colloid or by direct i.v. administration of the SPIO suspension (in vivo labeling).

Although uptake of SPIO and USPIO in mononuclear cells causes a transient oxidative stress, it does not affect, even at high iron loads, in vitro cell survival, proliferation, mobility, and phagocytic properties [47, 48].

It is useful here to remember that iron oxide phagocytosis is not a function restricted to a specific phenotype of mononuclear cells. In particular, as part of a continuum of mononuclear cells’ behavior [49], both pro-inflammatory (so-called M1, typically induced by exposure to IFN-γ and TNF-α) and anti-inflammatory “M2” (induced by IL-4 or IL-13) phenotypes share phagocytic properties.

As a consequence, both these phenotypes present a sizeable uptake of colloids, although CD14++ CD16—(typically “pro-inflammatory”) monocytes have shown in vitro more efficient phagocytic properties toward iron oxide nanocolloids, compared to CD14++ CD16+ (anti-inflammatory) monocytes, as tested with both SPIO and MNIO [50].

In addition, an induction of a shift towards an anti-inflammatory phenotype following SPIO uptake has been suggested [51].

Accordingly, non-specificity of these particles, as pertaining to the pro- or anti-inflammatory phenotype of the monocytes, must be taken into account when interpreting the results of studies based on this approach to cell labeling.

Alternatively, cells can be selectively labeled by superparamagnetic particles functionalized with specific antibodies.

### In-vitro labeling through phagocytic cell properties

Advantages of in vitro labeling are the high load of iron per cell that can be achieved using this approach, and the specificity of the labeling (although in principle cell death subsequent to i.v. administration can still result in the presence of free iron colloids in the bloodstream).

Main in vitro labeling strategies included the use of dextran-coated SPIO complexed to poly-l-lysine [52], the incubation with protamine sulfate as transfection agent [52]*, or electroporation [53].

Given the higher complexity of these strategies, compared to the direct in vivo i.v. administration of superparamagnetic colloids, relatively less studies have been carried out using this approach. However, using these strategies, detection in the spinal cord of mice with experimental allergic encephalomyelitis (EAE, a preclinical model of MS) of lymph node cells sensitized with the proteolipid protein antigen used for EAE induction [54], or of myelin basic protein-specific T-lymphocytes [52], labeled in vitro using SPIO, has been achieved on T2*-weighted images.

Incubation of MNIO with spleen-derived mononuclear cells in a mouse model of transient middle cerebral artery occlusion has also been used, allowing to track blood-borne inflammatory cells to the periphery of the infarction [55].

In-vitro labeling strategies have been used so far only in preclinical studies of neuroinflammation, although they have been proved to be safe in humans [56], and have been used to image cell migration to cutaneous inflammation sites. Currently no studies of neuroinflammation in humans are reported using this approach.

### In-vivo labeling through phagocytic cell properties

The ability of the monocyte/macrophage cell population to phagocytose in vivo nanoparticles has been used to label these cells by i.v. injection of iron nanocolloids to track by MRI cell migration following neuroinflammatory phenomena in various rodent stroke models.

The labeling efficiency of iron nanocolloids following i.v. injection is essentially a function of the phagocytic potential of the cells. Among circulating cells, mainly blood monocytes (and to a much lesser extent neutrophils) show a significant uptake.

Lymphocytes are essentially not labeled using this approach due to their low spontaneous phagocytic activity and limited cytoplasmic volume.

Phagocytic cells typically reach the infarct core on days 2–4, coming from the periphery of the lesion, and leave by 1 week post stroke, a temporal pattern confirmed by experiments carried out in the photothrombotic stroke model (providing standardized localized infarct lesions without penumbra) [57].

Bone-marrow cells labeling in chimeric organisms [58] has shown that microglial activation precedes migration from blood of machrophages.

In the transient MCAO stroke model in rats, USPIO uptake has been shown in the infarcted region [59, 60], in agreement with histology. In addition, peri-infarct USPIO uptake and spread to the contralateral hemisphere has been shown in the same model [61], again in agreement with histology data.

Interestingly, at later time points a mismatch between the presence at histology of macrophages and the lack of USPIO labeling has been found in photothrombotic stroke model, suggesting a lack of phagocytic activities at these later time points.

Despite the advantages represented by the simplicity of the in vivo labeling procedure, and by the lack of manipulation of the mononuclear cells, which may allow a more natural behavior of these cells, different potential confounding factors should be still kept in mind when interpreting results obtained using this approach.

In particular, somewhat conflicting results have been reported when assessing the route followed by iron colloids, especially of smaller size, following i.v. injection, to reach the inflammatory sites, where they are detected in phagocytic cells at histology. Indeed the possibility that these particles cross a damaged BBB independent of cell trafficking, and are subsequently captured by resident microglia, must be carefully considered. Enhancement related to dextran-coated iron oxide nanocolloids has been in fact shown following BBB disruption by freezing or by osmotic shock, although in the latter case only MNIO particles (20 nm) crossed the basement membrane, as opposed to SPIOs (200 nm) [62], so that the size of the particles seems to play a major role here.

Consistent with this pattern, USPIO enhancement is seen already within hours after photothrombotic stroke, as opposed to monocytes labeled in vitro with SPIO [63], supporting the hypothesis of a non-specific uptake from resident microglia, following diffusion made possible by BBB disruption.

Mononuclear cell migration to the stroke site has been studied also in humans using i.v. injection of iron nanocolloids. USPIO injection 7 days after the stroke has shown a similar pattern of USPIO uptake in the ischemic region, interestingly unrelated to Gd-DTPA enhancement [64, 65], thus suggesting, at least under these specific clinical conditions, a relative independence of the colloid uptake from the disruption of the BBB.

These same considerations apply to the EAE studies carried out using nanocolloids, as early (within 1 h from injection) uptake of USPIO has been shown in rats with EAE in cerebellar regions, which are characterized by extensive BBB breakdown detected at Gd-DTPA-enhanced T1-weighted images [66], clearly pointing at extravasation as uptake mechanism. However, foci of USPIO uptake unrelated to BBB breakdown, and corresponding at histology to phagocytic leukocytes clusters, have been shown at later time points [67, 68].

In addition, in EAE the extension and intensity of USPIO uptake correlated with phagocyte infiltration, and in turn with demyelination and axonal loss [69], indirectly suggesting a clinical relevance of the phenomena probed by USPIO.

Of note, in interpreting these results, beside wash-in mechanisms, the role of a possible wash-out of USPIO from the brain, either by cell migration or free diffusion, typically to cervical lymph-nodes, should also be considered [66].

Finally, a lack of a direct relationship between USPIO uptake and DTPA-Gd enhancement has been shown [70] also in MS patients, thus indicating a possible complementarity of the information provided by these two MRI parameters.

All together, these data suggest a time-dependent specificity of colloid uptake related to the simultaneous presence of BBB breakdown (allowing the colloid to reach freely the interstitial space) and of cells with phagocytic activity (either resident or blood-borne), these conditions being both present in the very early phases after stroke. At later time points, the dissociation between BBB breakdown and USPIO uptake suggests more specific mechanisms of uptake, secondary to phagocytose of these molecules by blood monocytes, which then cross an intact, or only mildly permeable, BBB.

### Specific leukocyte labeling

Direct in vivo labeling of immune cells, including CD4, CD8, and machrophage-specific epitopes has been obtained using USPIO-labeled antibodies [71], allowing the in vivo imaging in mice of immune cell migration to the inflammation sites in EAE and viral encephalitis.

However, although a superior specificity can be in principle obtained using this approach, it must be still considered that, despite their different origins [43], specific markers are not currently available that allow to reliably discriminate microglia from blood-borne inflammatory cells reaching the site of inflammation so that superiority of this approach compared to phagocytosis-based labeling remains to be proved.

### Leukocyte functional imaging

Gd-bis-5-HT-DTPA (MPO-Gd) is a molecule activated by myeloperoxidase (MPO), a proinflammatory oxidative enzyme secreted by activated neutrophils and monocytes at inflammatory sites. MPO’s effect on MPO-Gd results in its oligomerization and increased binding to proteins, which both determine a T1 effect, allowing its detection in T1-weighted images [72, 73].

T1 shortening in neuroinflammation areas using this molecule has been proved to be specific by its negativity in a MPO-knockout stroke mice [74].

MPO-Gd has been used to assess neuroinflammation in stroke [74] and EAE [75], as well as to detect neuroinflammation following treatment by oncolytic virus in a rat model of glioma [76].

In EAE in particular, it has shown a higher sensitivity compared to BBB integrity tracers (Gd-DTPA). Of note, a MPO-inhibitor has been used as experimental drug in EAE mice, showing a reduced inflammatory and demyelinating phenomena in treated mice [75], suggesting a possible specific role of this approach for specific patient stratification and future therapy monitoring.

Overall, no fully satisfactory method to image leukocyte migration in humans by MRI is currently available. While in vivo cell labeling by i.v. injection of iron colloids suffers from limited specificity, and only a few pilot studies of neuroinflammation in MS and stroke have been reported, no method to label specific leukocyte classes has reached the clinical use, also due to safety issues that still need to be addressed.

In vitro labeling, which allows specific labeling of leukocyte subclasses, could also provide very high iron loads allowing to achieve single cell detection, comparing favorably with currently available nuclear medicine techniques. However, no brain studies in humans are currently available using this approach.

It should also be noted that MRI studies using leukocyte-labeling techniques are not inherently quantitative. Indeed, iron oxide colloids have been used so far in human studies essentially as indicators of “on/off” phenomena, such as inflammatory activity in MS lesions, a pathology where the endpoint for therapies is the reduction of the number of “active” plaques, instead of the reduction of the overall “intensity” of the inflammatory activity.

Finally, these techniques do not allow in general to compare the results over time, as several factors, not limited to the variable labeling efficiency of cells, may vary significantly over time, independently from inflammatory phenomena (e.g., changes in signal of the background due to lesion evolution may change the detectability of USPIO accumulation, especially if hemorrhagic phenomena are present).

On the other hand, nuclear medicine methods for leukocyte imaging which have been used to image neuroinflammation, such as leukocyte labeling by 99mTc or ^18^F-FDG, also suffer, beside the limited spatial resolution, from several shortcomings. These include the presence of non-specific signal from the radiolabel eluted from the cells (a weakness common to the MRI techniques), or the limited timeframe for acquisition following the injection due to the short half-life of isotopes used in PET and SPET imaging.

## Alterations of the interstitial tissue composition

Diffusion-weighted Imaging is a technique that exploits MRI exquisite sensitivity to motion to provide maps of the bulk motion of water molecules due to diffusion phenomena in biological tissues [77], thus providing an in-depth analysis of the microstructural features of the tissue [78].

Selective diffusion reduction along specific directions occurs in anisotropic tissues, such as the white matter, in which diffusion is not equal in all directions, due to the presence of impermeable or semipermeable walls, such as parallel cell membranes and myelin sheaths [77].

The use of sequences probing water diffusion along six or more diffusion directions allows to calculate a full diffusion tensor (diffusion tensor imagig—DTI), providing a full-3D description of the water diffusion properties. From DTI, maps of the apparent diffusion coefficient (ADC) and fractional anisotropy (FA) can be calculated [79]. ADC is an estimate of the mean magnitude of water movement (independent of the direction), while FA is an estimate index of the anisotropy of water diffusion [77].

BBB permeability alterations invariably result in a change in the interstitial space composition, with extravasation of macromolecules and onset of vasogenic edema [80], which in turn determines an increase in ADC and a reduction in FA, readily detected by DTI.

Diffusion-weighted imaging has allowed to study the presence and time course of vasogenic edema in several neuroinflammatory conditions (e.g. infections, traumatic brain injury, stroke, demyeliniating diseases), as well as the mechanisms underlying its resolution [81].

More recently, post-processing algorithms have been developed to derive, from the DTI data, maps of the diffusion properties of tissues, removing the diffusion changes due to the amount of free water in the voxel [82]. These methods have allowed detecting and characterizing subtle diffusion alterations, possibly linked to chronic inflammatory phenomena, in the brain of schizophrenic patients [83, 84].

Additionally, DTI data allow calculating separately maps of the diffusivity along the main axis of diffusion (longitudinal diffusivity) and along the plane perpendicular to this axis (radial diffusivity). Longitudinal diffusivity reflects water motion along the axons, mainly dependent on the presence and viability of axons, while radial diffusivity is considered influenced mainly by cellular barriers, and thus essentially by the integrity of myelin sheaths [85].

Accordingly, changes in these two parameters are used to assess the relative contributions of axonal loss and demyelination in determining diffusion changes in the brain in several human neuroinflammatory pathologies (e.g. in MS [86]).

Alterations of the microenvironment in terms of macromolecular composition and hydration state, resulting from BBB permeability alterations and activation of proinflammatory pathways in the brain, can be explored also by magnetization transfer (MT) imaging, which exploits the exchange of protons between the free water and the macromolecule compartments [87].

MT is probed by acquiring a T1-weighted volume preceded by an off-resonance RF pulse that excites selectively the protons bound to the macromolecules. Part of the magnetization conferred to macromolecules is then transferred, through magnetization interactions (dipolar and chemical exchange) to the pool of the “mobile” protons (those of the free water in tissue) resulting in a reduction in signal. MT is assessed quantitatively through the MT ratio (MTR, the difference between the signals obtained with and without the off-resonance RF pulse, divided by the signal obtained in absence of the off-resonance RF pulse).

As MT depends on the relative presence of the two pools in the voxel, changes in the water content due to inflammation result in a change of MTR [88].

Although main studies using MTR to assess neuroinflammation have been carried out in MS [88], MT has demonstrated to be sensitive also to more subtle neuroinflammatory phenomena, including the effects of systemic inflammation on the brain both in preclinical studies [89] and in humans [90].

Overall, both DTI- and MT-derived measures have shown a significant sensitivity to occult brain damage in MS. However, it should be highlighted here that both these techniques are non-specific (being variously influenced by axonal loss, BBB permeability alterations, inflammatory cell infiltrate, and demyelination). As a consequence, only few studies have included these measures when defining secondary endpoints in clinical trials of new disease-modifying treatments for MS, and their use in routine monitoring of MS patients is extremely limited [91].

## MRS markers of neuroinflammation

MRS studies of primarily inflammatory (e.g. MS) and infectious diseases of the CNS suggest that neuroinflammation can result in elevated levels of myo-inositol (mI, an organic osmolyte present in glial cells, participating in astrocyte volume regulatory mechanisms [92]) and choline-containing compounds (Cho) [93].

mI, in particular, has been shown in vitro to be a selective glial marker in rat brain tissue extracts [92], with selective expression of mRNA for sodium-dependent myo-inositol transporter in astrocytes [94].

mI increases, which have been detected both in the active [95] and non-active phases of MS [96], are present already in the early stages of the disease [97], and are not limited to MS lesions, but involve also normal-appearing white matter [98, 99].

Viral infections are also characterized by increased mI. In particular, HIV+ patients show increased mI [93], while in chronic hepatitis C infection, a condition linked to neuropsychological symptoms and cognitive impairment, a significant increase in mI in basal ganglia has been shown [100, 101], correlating to ^11^C-(R)-PK11195 uptake at positron emission tomography, consistent with significant microglial/brain macrophage activation.

Of note, MRS allows to simultaneously assess the degree of neuronal dysfunction/loss, concurrent with (or due to) neuroinflammation by measuring *N*-acetyl-aspartate (NAA), a selective marker of mature neurons. Accordingly, in all the above conditions NAA decreases have been also monitored allowing to assess in an integrated manner the relationship between neuroinfammation and neuronal loss [93].

However, these changes are non-specific, as mI can be elevated in non primary inflammatory conditions, such as hyper-osmolar states (e.g. hypernatremia, renal failure, and diabetes [102, 103]), as well as in alcohol-dependent patients [104], possibly due to alcohol-induced hyper-osmolarity.

Also body-mass index in obese [105] and non-obese subjects [106, 107] correlates to mI levels, which in turn demonstrates a strong relationship with white matter fractional anisotropy [107], possibly reflecting an effect of neuroinflammation on white matter microstructure.

Furthermore, mI increases with age in normal brain have been demonstrated, possibly due to the simultaneous presence of demyelination and glial proliferation [108].

On the other hand, increased choline has also been detected in several non-primarily inflammatory brain disorders, including brain tumors [109] stroke [110], epilepsy [111], traumatic brain injury [112, 113], and HCV [100, 114]. In these pathologies, Cho increase has been variously interpreted as reflecting in turn products of membrane degradation, angiopathy, edema, or energy failure.

In summary, MRS has shown a sufficient sensitivity for assessing neuroinflammatory disorders, and has been applied in MS, neuroviral infections, the main markers of active neuroinflammation being increased myo-inositol, choline, and total creatine [93]. Although its limited specificity hampers its potential in terms of differential diagnosis, suitable applications have been found for longitudinal monitoring, especially in neuroviral diseases, while MRS-derived measures of neuronal loss (NAA reduction) have shown a clear potential in terms of both prognostic value and assessment of the effects of treatments in MS [115].

## Conclusion

With the growing interest in the role of neuroinflammation, also in pathologies not traditionally considered as primarily neuroinflammatory (e.g. Alzheimer’s disease [116] or psychiatric disorders [117]), non-invasive techniques to study in vivo the complex cascades of events involved in different aspects of neuroinflammation are increasingly required.

In MRI, the advantages represented by the large availability of the technique, and by the lack of exposure to ionizing radiations, are coupled to the possibility to assess simultaneously different features of neuroinflammation, ranging from BBB breakdown and interstitial microenvironment changes, to cellular infiltration and gliotic reaction.

As a consequence, as more and more aspects of neuroinflammation become accessible to probing by MRI/MRS, an increasing number of studies is carried out taking advantage of the complementarity of the information provided by this technique.

However, especially when compared to nuclear medicine techniques, that allow a sensitive and specific detection of microglial activation [2], specificity of the MRI techniques remains an issue.

From this standpoint, a further advancement can be expected following the recent introduction of hybrid PET/MRI scanners, which allow to cross-correlate the different datasets avoiding time-dependent variability.

Furthermore, the simultaneous acquisition of PET and MRI allows to co-register easily and in an operator-independent manner the PET, which lacks anatomical landmarks when PET neuroinflammation tracers are used, with the MRI.

In preclinical studies, the possibility to carry out longitudinal studies assessing simultaneously perfusion, BBB permeability, and infiltration from monocytes using MRI, and microglial activation using PET, is expected to help clarify the time course and the complex interactions of inflammatory phenomena in primarily neuroinflammatory diseases and in stroke.

In clinic, the accurate co-registration with structural studies achievable with hybrid scanners can improve our sensitivity when very limited tracer uptake is present, provided it occurs consistently in the same anatomical structure. To date, PET/MRI studies have been used to demonstrate subtle but significant glial activation, correlating with pain severity, in specific brain regions in patients with chronic back pain [118] and in the motor cortices and corticospinal tracts of patients with amyotrophic lateral sclerosis [119].

Despite the abovementioned limitations, in vivo neuroimaging methods help unraveling the multi-faceted role of neuroinflammation in several CNS diseases, including pathologies once considered exquisitely neurodegenerative, and the currently ongoing transition from pre-clinical to clinical applications of these techniques has the potential to introduce significant changes in the management of these diseases.
